# Making Partnerships Work: Proposing a Model to Support Parent-Practitioner Partnerships in the Early Years

**DOI:** 10.1007/s10643-021-01181-6

**Published:** 2021-04-18

**Authors:** Maria Kambouri, Teresa Wilson, Myria Pieridou, Suzanne Flannery Quinn, Jie Liu

**Affiliations:** 1 Institute of Education, University of Reading, Reading, UK; 2 Faculty of Wellbeing, Education and Language Studies, The Open University, Milton Keynes, UK; 3 KOMPAN Play Institute, 605 W Howard Ln - Suite 101, Austin, TX 78753 USA; 4 The UCL, Institute of Education, London, UK

**Keywords:** Parent and practitioners or teachers collaboration, Froebel's pedagogy, Homeschool relationships, Working in partnership with parents, Developing early years practice

## Abstract

Building on Froebelian principles that highlight the importance of family and community, this study explored the importance of collaboration and communication as part of a two-way dialogue. The aim was to identify the key characteristics of a model that would encourage interest and commitment to partnerships from both parents and practitioners. The idea of such partnerships has a solid theoretical background and is supported both rhetorically and by legislation by the Department of Education. However, research has shown that practice often falls short of the ideal, due to reasons such as the managerial discourse that constructs parents as potential consumers and the challenges faced when performance is prioritised over creativity. As part of the study, we employed a mixed methods approach and encouraged parents and practitioners to work together by participating at two sessions with families and children. The sessions provided parents and practitioners with space and time to explore the issue of working in ‘partnership’. After careful consideration of ethical issues, data were collected using pre and post-session questionnaires with all participants, as well as face to face interviews with some of them. Findings indicate that both parties need to invest time and recognise that ‘effective partnership’ is a two way process which requires engagement and dialogue to be able to develop meaningful relationships of trust. The findings were used to develop the ‘CAFE' partnership model which incorporates those elements considered important to facilitate the development of partnerships between practitioners and parents. The CAFE model addresses the gap in the literature in terms of unpicking the key features of a partnership approach, as captured through the lived experience of both parents and practitioners. It also contributes to deepening the understanding of the applications of Froebelian principles in contemporary contexts and the ways in which they can encourage high quality early childhood development and education. Future research should explore how this model could be used to evaluate existing practice and guide the development of current partnership policies and approaches.

## Introduction

Parents^1^ are their children’s first educators and they share knowledge with their children through engagement in everyday activities and play, and they continue to support their children’s learning once the children enter institutional settings, such as nurseries or child-care (Goodall & Montgomery, 2014). Once children enter institutional child-care or educational environments, the scope of their experiences and relationships with other people widens (Desforges & Abouchaar, 2003). At this point, it is key for parents to continue to be part of their children’s education (Goodall & Montgomery, 2014) and, therefore, developing parent-practitioner partnerships is crucial in facilitating and encouraging this. This issue has risen in importance as the relationship between parents/carers, children, and their educators has been altered notably during the COVID-19 pandemic, when educational settings have depended increasingly on parents to continue the education of their children (Department for Education (DfE), 2021; Montacute & Cullinane, 2021; Wilson & Waddell, 2020).

Recognising the benefits, whilst at the same time acknowledging the limitations of such partnerships is important, especially due to the possibility that one partner may hold more power than the other in the relationship (Goodall & Montgomery, 2014; Vincent, 1996). The nature of parent-practitioner relationships has been the subject of several investigations, where both the definition of the relationship and the meaning of the terms ‘involvement’ and ‘partnership’ have been subject to interpretation (Barton et al., 2004; Cottle & Alexander, 2013; Desforges & Abouchaar, 2003; Devine, 2004; Goodall & Montgomery, 2014). There are a significant number of factors to consider in what has been called a ‘messy web of interactions’ (Goodall & Montgomery, 2014, p. 400), which might include both the location and the level of engagement between the parent and the practitioners at the child-care or educational setting. Nevertheless, there is a lack of clarity in the literature about what exactly is meant by ‘parental involvement’ and ‘parental engagement’, as the terms appear to often be used interchangeably, even though they may have distinct interpretations. It appears that involvement and engagement are concepts that refer to partnership or are aspects of partnership (Barton et al., 2004; Cottle & Alexander, 2013; Goodall & Montgomery, 2014). For example, as part of a continuum (Goodall & Montgomery, 2014), the partnership could be encouraging achievement and valuing education, or it could be engagement in school processes or attending parenting classes. In most cases, parental involvement has a fluid meaning but, in every case, there is an element of interaction between parents and practitioners in early years settings. For this study, the concept of ‘partnership’ between parents and early years practitioners is used, with the focus towards working together for children’s best interests and finding a common ground for involving parents, while maintaining a clear understanding of issues around roles and power, barriers and enablers of teacher-parent collaboration. Previous research has shown that power dynamics may divide the experiences of parents or teachers and can result in some feeling less engaged or marginalised (Pieridou, 2013; Ware, 1994); therefore, the concept of ‘partnership’ was highlighted throughout the project due to its emphasis on collaboration and mutual respect within the structure of teacher-parent conversations (Pieridou, 2013).

Effective and meaningful collaboration and parental involvement in early education are known to be an essential part of parent-practitioner partnerships, and meaningful parent-practitioner partnerships require mutual respect and recognition of the contribution each key agent makes towards children’s development (Baum & McMurray-Schwarz, 2004). Despite the plethora of evidence and general consensus about the importance and multiple benefits of parent-practitioner partnerships (Murray et al., 2015; Nachshen, 2004; Rouse, 2012; See & Gorard, 2013; Sylva et al., 2004; Turnbull & Turnbull, 2001; Vincent, 1996; Wheeler et al., 2009; Wilder, 2014; Wilson, 2015), there is little known about how this can be achieved in practice, and there is no model available at the moment to support both parents and practitioners, as well as researchers and policy makers, when unpacking the key characteristics of an effective parent-practitioner partnership model.

The aim of the study presented in this paper is to address this need by identifying the characteristics of a parent-practitioner partnership model, developed specifically to help early years practitioners and parents of young children, aged between 3 months and 5 years old, to work together when supporting children’s holistic learning and development. The study employed a mixed method approach and engaged both parents and practitioners in order to develop a flexible parent-practitioner model that builds on their experience and recognises the complexity of the relationship and the positive impact a constructive partnership can have on children and their learning outcomes, both in their life and overall well-being (Sylva et al., 2004; Turnbull & Turnbull, 2001; Vincent, 1996; Wheeler et al., 2009; Wilder, 2014). At the core of the study were the elements of contemporary Froebelian pedagogy, specifically the importance of knowledgeable and appropriately qualified staff, and the need for early years settings to be an integral part of the community, working in close partnership with parents and other skilled adults (Froebel c1826 trans., 1912). The study is also contextualised by an awareness of the potential power dynamics in teacher-parent relationships. Therefore the design and implementation of the partnership sessions included activities that welcomed the participants’ experiences and respected their voices, along with dedicated discussions on mindfulness and building relationships of trust, that were aimed to promote balanced participation.

In the paper, we discuss the importance, as well as the limitations, of developing parent-practitioner partnerships and we argue that both the parents and the practitioners need to be empowered in this process, to engage with each other effectively. The methodological approach for this study is novel, as previous studies have tended to rely on one method rather than mixed methods, and to the best of our knowledge, no previous study has involved parents and practitioners working together as part of the research methodology. In that sense, this paper is original not only in terms of developing a partnership model, but also in its consideration of power dynamics; by choosing to emphasise a balanced participation of parents and practitioners, so that the partnership model is engaging and based on valuing both parents’ and practitioners’ perceptions and lived experiences. This allows for the possibility to try and reduce the gap within the literature and develop meaningful links between theory and practice.

The partnership model can, therefore, offer an avenue in overcoming some of the barriers of parent-practitioner partnerships by developing relationships of trust and empowering parents to recognise the significance of their role. The model is based on the value of parents and practitioners learning together and developing a supportive environment around the child, that will help the child flourish, building on Froebel’s principles and their application in contemporary contexts (Froebel c1826 trans., 1912). Finally, the paper also contributes to the literature with a novel approach of applying Froebel’s theory, and specifically the distinct Froebelian principles of interconnectedness of people, in a contemporary context, which allows further exploration of Froebel’s contribution in today’s early childhood education.

## Literature Review

### Regulatory Framework

Parent-practitioner partnerships can be seen as a current priority in educational practice. It is framed by formal requirements in England, contained within the Statutory Framework of the Early Years Foundation Stage (Department for Education (DfE), 2017); the Teachers’ Standards (DfE, 2011); Early Years Teacher Standards (National College for School Leadership (NCTL), 2013), and the Ofsted early years inspection framework (Office for Standards in Education, Children's Services and Skills (Ofsted) 2018. In the recent White Paper, Early Excellence Everywhere, it was stated that “…we must do more to ensure all parents have a more significant voice in schools” (DfE, 2016, p. 17). When practice is assessed through the Ofsted inspectorate, evidence is sought that parents are actively encouraged by practitioners to engage in their children’s development (Ofsted, 2018). The Department for Education and Skills (DfES, 2007, p. 6) also notes that “there is clearly significant public interest in making it as easy as possible for parents—fathers and mothers—to engage as partners in their children’s learning and development from the earliest age as early as possible”.

Enhancing parent-practitioner partnerships is also of interest globally. The development and maintenance of positive relationships between parents and practitioners appears in policy and guidance from the European Commission (2014), in the Head Start Performance Standards in the US (US Department of Health and Human Services, 2016), and is noted as a ‘quality area’ within the National Quality Framework in Australia (Australian Children’s Education and Care Quality Authority, 2012). The New Zealand early years curriculum, *Te Whariki.* is underpinned by its principle of partnership with parents (Ministry of Education, New Zealand, 2017). It can be seen that engagement with this concept is widely shared, crossing cultural boundaries. In the following literature review, the nature of the partnership itself and both the benefits and the challenges of this particular relationship will be explored.

### Unpicking Parent-Practitioner Relationships

Before exploring the established benefits and challenges to effective parent-practitioner partnerships, the extent of what would be classified as partnership activities should be explored, as well as the degree to which the roles in the parent-practitioner partnerships are equally weighted (Rouse, 2012).

Despite the unchallenged acceptance of the importance of teacher-parent partnerships, research indicates differing interpretations of partnership by both parents and practitioners, which extends to ‘problematic’ experiences of partnerships (Hodge & Runswick-Cole, 2008). These interpretations may be exacerbated by the models of parental involvement, which have been critiqued as “representing a restricted view that fails to account for diversity in parental involvement practices” (Daniel, 2015, p. 120). In other words, one challenge in defining parent-practitioner partnerships is being clear about the range of activities that count as relevant, including those that are less visible as they take place away from the setting. For example, Epstein’s model of parental involvement (Epstein et al., 2009) presents a typology of behaviours associated with improved outcomes, which includes volunteering and decision-making, communicating, collaborating with the community, and learning at home. However, in data produced by the Organization for Economic Cooperation and Development (OECD, 2017) the impact of parents on their children’s outcomes as they progress through school is not restricted to engagement with setting-based activities, but also includes simple social situations, such as taking an interest in children’s development whilst having a meal together. The partnership model discussed here aims at valuing parents’ existing engagement and letting parents know that what they are already doing is great, as this can be very rewarding and can encourage further commitment for those parents who struggle to engage in the life of the setting because of work or other commitments (Campbell et al., 2016; Daniel, 2015). It may be that a starting point for optimising the parent-practitioner relationship is through the celebration of what parents are already doing, in a relationship based on family-centred practice, where “empowerment is a central component” (Rouse, 2012, p. 22), like the partnership model of Davis et al. (2002), where more individualised relationships and methods of communication develop, giving parents confidence in their ability to have an impact on their children’s development.

This is also linked to one of the most common barriers faced in parents and practitioners’ partnerships; the possibility of power imbalances in the relationship. Research indicates that practitioners usually enjoy more power in the relationship, based on their sense of professional knowledge of child development or pedagogy; in some instances, practitioners may regard parental knowledge about child rearing as naïve, or not based on ‘expert’ knowledge of child development principles (Pieridou, 2013), while parents report feeling passive during decision making meetings (Murray & Mereoiu, 2015). In effect, parents argue that their holistic understanding of their children’s characteristics is de-valued or neglected (Pieridou, 2013, Hodge & Runswick-Cole, 2008), despite the fact that “parents are the only unpaid volunteers involved in a sea of high-paid specialists, who have a full diachronic image of the child, when different professionals come and go” (Phtiaka, 2008, p. 123). Unfortunately, even though parents’ participation has been subject to legislation during the last decade, evidence (both in the UK and in other European countries) indicates that the procedures and strategies to develop partnerships remain vague, and the reality of partnerships between practitioners and parents is still far from ensuring an ideal and collaborative ethos (Barnes, 1994; Pieridou, 2013; Zoniou-Sideri & Nteropoulou-Nterou, 2008).

In addition to the above, some would argue that training specifically related to working with parents is in short supply in early years settings and could be used to enhance communication between parents and practitioners (Goodall & Vorhaus, 2011). In Wilson’s research (2015) one practitioner noted that ‘If you’ve got a fragile partnership only one harsh comment can potentially turn the relationship around for a long time’ (p. 108) and suggested that training for all workers would be advisable. An exploration of the literature related to perceptions of partnership can be revealing in terms of discussions of powerbases, decision-making, and professional expertise, as well as to questions of whether parents should also have training to hold positions of authority in the setting (Foot et al., 2002).

### The Importance of Parents and Partnerships

It is widely recognised that the parent-practitioner partnership is not only important because of regulations and requirements: these regulations have been introduced in response to evidence of their efficacy. The development of positive relationships between parents and practitioners can lead to wider opportunities to signpost and support families whose children have conditions potentially leading to long term disadvantage (Khan, 2014; Wilson, 2015). As Khan (2014) commented, professionals and families have brief and important opportunities to identify and activate support for children with insistent behavioural problems.

Existing research recognises the critical role played by parents in terms of child development. Studies over the past two decades have provided detailed information about the areas where this development is particularly clear. Longitudinal studies such as the ‘Effective provision of pre-school education (EPPE) Report’ (Sylva et al., 2004, p. 40), highlight the importance not only of parental engagement in a setting, but of the potential of the “home learning environment”, which “exerts a significant and independent influence on attainment at both age three years and later at the start of primary school”. Other research has highlighted the impact of partnerships with parents or the home learning environment on social and emotional development (Wheeler et al., 2009), language and literacy development, mathematical language development (Evangelou et al., 2009), and school readiness, in addition to benefits associated with pre-school (Melhuish et al., 2008).

The EPPE findings highlight the impact of parent-practitioner partnerships on social/behavioural and cognitive development. The EPPE findings (Sylva et al., 2004) support the principle of practitioners working with parents to promote activities and the development of a home environment that provides rich learning opportunities. However, caution should be applied to the reasoning that all parent-practitioner partnership research can demonstrate rigorously evidenced outcomes for children, because it is generally difficult to isolate the parent-practitioner partnership and the impact it has had (See & Gorard, 2013; Wilder, 2014). See and Gorard (2013) noted that interventions are most likely to succeed when they are aimed at young children, and involve parents and staff meeting regularly in an institution, with parental training, ongoing support, and cooperatively working with teachers. They also highlight the challenges of pinpointing the impact of the involvement in terms of its effects on children’s academic achievement.

Additionally, it has been argued that the promotion and establishment of partnerships between parents and professionals can be the means for achieving inclusive schools (Barton and Amstrong, 2001; Boutskou, 2007; Pieridou, 2013). Boer et al. (2010, p. 166) argue that when parents of children with and without disabilities are positive towards inclusive education, then teachers and support staff “are more inclined” to implement it. Phtiaka (2004) adds that parents’ knowledge and skills can enhance children’s education, particularly when this is done in close collaboration with teachers and practitioners. Parents hold a prominent role in realising good quality and community-based education for all (Vislie, 2003), as they can participate in the design and implementation of inclusion programmes and also be actively involved in the decision-making process of their children’s education (UNESCO, 1994), something in line with the Salamanca Statement’s need for “decentralization and local-area-based planning” (UNESCO, 1994, p. 38).

A recent report by Sutton Trust (Sammons et al., 2015) examined ways of raising the educational attainment of children from disadvantaged backgrounds and stated that early years experiences up to the age of seven, along with better home learning environments and strong parent-practitioner partnerships, provide a significant boost in attainment at the age of eleven, and help to counteract disadvantage. As with many practices, when parent-practitioner relationships work well, the participants enjoy high levels of trust and collaboration. Unfortunately, the barriers associated with these partnerships can limit the effectiveness of the practice.

## Barriers and Limitations to Parent-Practitioner Partnerships

The review of the literature reveals both the importance and complexity of partnerships between practitioners and parents. A common cause of discrepancies and disagreements concerns the different ways in which key agents define partnerships and how they can be utilised in specific settings (Pieridou & Phtiaka, 2011). Legislative attempts to regulate partnerships do not always deliver a clear understanding of how these partnerships can function in practitioners’ and parents’ everyday lives, which reminds us that policies are not only about what is written, but also about interpretation and enactment (Ball, 1994).

Just as the challenge of defining parent partnership is complex, so too is the process of identifying the factors affecting the quality of the relationship (Cottle & Alexander, 2013). There are a number of barriers that can impede the development of positive relationships between early years practitioners and parents of young children. A frequent barrier can be found in communication when English is an additional language: language barriers make communication more challenging, and therefore reduce opportunities to become involved (Dyson 2001, cited in Murray et al., 2015). Goodall and Montgomery (2014, p. 400) note that there are also challenges derived from assumptions made about parents who “do not share the same world views” or who do not understand the “rules of the game” (Lareau et al., 2016, p. 279). Other issues, such as parental health problems can limit visits to school, as may long working hours, shift work, and children being dropped off and picked up by child carers (Goodall & Montgomery, 2014; Hughes & MacNaughton, 2002). Indeed, Daniel (2015) notes that very often the reasons for poor engagement with a setting are too complex to categorise easily, but what is clear is the lower instance of participation from lower SES families, thereby potentially increasing disadvantage to those children.

Further barriers include the ‘othering’ of parents in a binary relationship with professionals (Hughes and MacNaughton, 2002), and their possible exclusion from a “hierarchical autonomy and well-established power structures” in educational settings (Ware, 1994, p. 339). This is particularly noticed when practitioners who are seen as ‘professionals’ or ‘experts’ work with parents in educational settings in order to evaluate, re-evaluate, or discuss the ongoing support given to children based on their individual needs. In these cases, children may be identified as having special educational needs or disabilities, or be coming from a diverse background and need additional support. Research in these cases has shown that practitioners promote the idea that they know what is best for children (Brown, 1999) and often disqualify or disregard parental knowledge; treating it as inadequate and unprofessional (Phtiaka, 2008; Ware, 1999).

Within the framework of this study, barriers will be investigated, as previous literature indicates this as an area needing additional investigation. Furthermore, it appears that these barriers can exist in combination or in isolation, and their appearance varies depending on the context. It is also clear that barriers to collaboration have existed in educational settings over a long period of time, and therefore long-term engagement and commitment from all key agents ss needed in order to make any discernible difference (Barnes, 1994; Zoniou-Sideri & Nteropoulou-Nterou, 2008). To add to this complexity, there is evidence indicating that many practitioners have had no training in working in partnership with parents (Wilson, 2015). Thus, there is currently a gap in understanding the reasons behind the lack of engagement in developing parent-practitioner partnerships. Neglecting the skills to develop the relationships can easily result in superficial, rather than trust-based, communication.

## Theoretical Framework

Our novel approach to parent-practitioner partnerships takes inspiration from the pedagogic principles of Freidrich Froebel, founder of the kindergarten in nineteenth century Germany. The legacy of Froebelian kindergarten pedagogy is seen in many contemporary early childhood education practices in Europe, North America, Asia, and Australia, where it has formed a foundation of ‘child centred’ practices and has supported high quality early care and education. Froebelian pedagogy is distinct, in that it is based on Froebel’s concept of ‘unity,’ which can be interpreted as a recognition of the interconnectedness or interdependence of people (particularly children, parents, and early years practitioners) with each other, and within an ecological and cultural system. Since Froebel’s kindergarten is considered to be a starting point in early childhood education, many contemporary early childhood programs share similar values with Froebel’s approach because they have grown out of this tradition. For example, in England, the EYFS recognises the importance of making decisions based on children’s interests (which may be considered a ‘child centred’ approach) and also recognises the importance of families in the lives of children. Both of these points are encompassed in a Froebelian approach but are also similar to other approaches in early education. What is distinct about a contemporary Froebelian approach is the concept of recognising not only the importance of families, but the striving for ‘unity’ in an understanding of how practitioners can work collaboratively with families, in the best interests of children. Froebel’s pedagogic principles rest upon the idea that parents and family members form the basis for a child’s understandings and interactions (Froebel c1826 trans., 1912). This requires both parents and practitioners to feel empowered in order to engage in the partnership. Empowerment in this case refers to the ability to be assertive while having self-efficacy and confidence in controlling resources and making decisions (Zimmerman, 1995). Turnbull and Turnbull (2001) agree that empowerment is an individual’s capacity to make decisions and solve problems, while Dunst and Dempsey (2007, p. 306) define empowerment as ‘the attitudes, knowledge, and behaviours associated with perceptions of control, competence, and confidence’.

When discussing family empowerment, Thompson et al. (1997) argue that empowerment is about individuals being able to determine their own future, and families having the appropriate information and problem-solving skills to deal with challenging situations. In the context of developing parent-practitioner partnerships in the early years, empowered individuals should be sufficiently confident to engage with each other and develop relationships of trust, requiring a degree of openness which might suggest vulnerability. However, being empowered would also mean that both parents and practitioners would be able to access knowledge, skills, and resources, enabling them to make decisions, have control, and support their children’s learning and development in a more positive and impactful way (Rouse, 2012; Singh, 1995). Being empowered is a key aspect of the parent-practitioner partnership, as it recognises the important role that both parents and practitioners play. Both parents and practitioners will enter the relationship with their own individual capabilities and needs, as well as the willingness to work together and the ability to develop and become more competent (Dunst & Dempsey, 2007; Rouse, 2012).

The idea of empowerment in the context of parent-practitioner partnerships has been discussed in the past, as part of the family-centred partnership model. However, this model suggests that practitioners are the experts and parents are in need of their expert support (Dunst et al. 1988). In this sense, the model presents practitioners as ‘help-givers’ and parents as ‘help-seekers’ (Davis et al. 2002; Nachshen, 2004; Rouse, 2012) which presents a context of inequality, with the professionals coming from a position of empowerment and the parents of relative disempowerment. Instead, in our approach we see parents and practitioners as equals and argue that settings have a responsibility to make themselves accessible to parents, while parents should also understand the value of their role. Our approach sees practitioners as the gatekeepers for their settings and should not take it for granted that parents are aware of their powerful role. Parents may need support to develop confidence or guidance in relation to the ways they can be involved in their children’s learning, particularly during times of crisis (e.g. the COVID-19 pandemic) (DfE, 2021; Montacute & Cullinane, 2021). It is easy to say, ‘parents chose not to be involved’, but with more active engagement and information to parents on their value, this might change.

Guided by the literature review and our understanding of the enablers and barriers to practitioner-parent partnerships, this study was designed to promote equal participation in practitioner-parent partnerships through using a set of principles that guided the development and realisation of the partnership sessions:

- *Neutrality of power* The partnership sessions were delivered outside the school settings,
- *Respecting voices* Participants (both practitioners and parents) shared their understanding of partnerships and identified their own goals using their experiences and the unique nature of their settings and lifestyles,
- *Reflection* Participants reflected on their pre-conceptions of partnerships and through sharing experiences and taking part in activities that re-examined how they could further develop their collaboration,
- *Praxis* During and after the implementation of the partnership sessions, participants were encouraged to apply their experiences, knowledge and understanding of partnership in their settings.
- *Voice* Participants shared their views and opinions in a safe, non-judgemental environment

## Research Methods

The aim of the study was to identify the key characteristics of a partnership model that would help to develop practitioner-parent partnerships in the interests of young children. The research questions that this study aimed to answer were the following:

1. What are parents’ and practitioners’ perceptions of working in partnership?
2. How do these perceptions change after taking part in the partnership sessions?
3. What are the key features of a parents and practitioners partnership model, based on the participants’ own lived experiences?

To examine these questions, a mixed research methods approach was employed to gain a deeper understanding of the perspectives of parent and practitioner participants, and to consider what is important when developing effective partnerships. According to Cohen et al. (2011), adopting a mixed methods approach allows researchers to collect more comprehensive data, providing results that have a broader perspective of the overall issue or research problem.

The research design involved the collection of both quantitative and qualitative data, which allowed us to develop an in-depth understanding of the participants’ views on key characteristics of an effective partnership model. To enable the identification of these key characteristics, the study focused on understanding parents and participants by exploring their ideas, perceptions, and experiences in relation to practitioner-parent partnerships. This involved (a) a pre-session questionnaire for parents and practitioners on partnership, to develop the sessions; (2) the partnership sessions delivered to both parents and practitioners; and  (c) a post-session questionnaire and interviews with parents and practitioners.

The parents and practitioners that participated in the study were selected from a larger sample, which included all early years settings within the Berkshire area. It is worth noting that the aim and philosophy of the study was to value all teachers’ and all parents’/carers’ experiences as equally important, and therefore the participants were not specifically selected based on any characteristics such as prioritising parents/carers who had children with or without special educational needs, or with one or more children. It is acknowledged that all experiences of parent–teacher partnerships are influenced by the circumstances parents/carers and teachers face in their daily-lives.

The research team used established links with local authorities and settings through the Reading University Partnerships within Berkshire, and identified six settings from which participants were invited for the study. The criteria for choosing the settings included:

- *Age of children:* The setting cares for children from birth to five years old
- *Location:* The setting is located within disadvantaged area in Reading, Berkshire (using Ofsted evaluations and local authorities’ reports as indicators)
- *Proximity:* The setting is in proximity to the workplace of the research team; this was taken into consideration for convenience in contacting participants and delivering the partnership sessions.

The first six settings on the Reading University Partnerships list were identified and invited to take part in the study. Four out of the six settings accepted the invitation, while two settings did not reply. Two additional settings were then invited, and both settings accepted, leading to a total number of six settings taking part. Ethical approval was gained by the University of Reading ethics committee, which follows the BERA (2011) ethical guidelines. Informed consent, anonymity, and participants’ right to withdraw at any time were ensured. All parents and staff members of the participating settings were invited and were informed that they could attend the sessions, without necessarily taking part in the data collection process of the project.

## Data Collection Methods

The first step of the data collection process included a pre-questionnaire, which was distributed to all parents and practitioners of the six nurseries selected for the study, inviting all parents and practitioners from the participating settings (250 parents and practitioners) to take part. The questionnaire aimed to collect parents’ and practitioners’ perceptions of partnerships as well as current practices, and did so mainly using closed ended questions and Likert scales. This was vital in terms of developing an overall understanding of the targeted population and their perspectives (Cohen et al., 2011) in relation to parent-practitioner partnerships.

The questionnaire was developed based on the key ideas identified during the literature review. They included a total of 36 questions, 12 of which aimed at collecting demographic information (such as gender, age, qualifications etc.). The rest of the questions covered topics such as the level of participants’ interest in parent-practitioner partnerships, perceived benefits and barriers to developing parent-practitioner partnerships, communication methods used by the setting, and methods used by the setting to develop partnerships. The pre-questionnaire was piloted with ten early years practitioners who did not take part in the main study, which helped us to revise and finalise the questions (Cohen et al., 2011). It was then distributed online (through Survey Monkey) and a total of 109 participants (89 parents and 20 practitioners) completed the pre-questionnaire, giving a response rate of 43.6%.

At the end of the pre-questionnaire, responders were invited to attend two partnership sessions, aimed at bringing parents and practitioners together, to discuss issues around developing effective partnerships. The partnership sessions were a key aspect of the data collection. They were voluntary and organised in such a way as to encourage the exchange of ideas, using a range of strategies to ensure that participants felt welcome and psychologically safe, in an inclusive environment that promoted respect and meaningful communication (Friend & Cook, 2013). The sessions provided parents and practitioners with the space and time to explore the issues surrounding working in ‘partnership’. They were delivered on campus, which offered a neutral ground for participants, rather than at the nursery settings (which might have been ‘home territory’ for the practitioners). Food and drinks, as well as childcare services were offered on site, which enabled 25 participants to attend (7 practitioners and 18 parents), while 28% of them (7 parents) used the childcare service provided. These parents particularly commented that they would have not have been able to attend the session if childcare services had not been provided.

The partnership sessions were framed around the Froebelian principles of importance of the relationship of every child to family and community (Brehony, 2009) and were also guided by participant responses to the pre-questionnaire. Each session consisted of discussion and activities related to specific topics, such as the benefits and challenges of working in partnership. The importance of effective communication was explored, looking at communication as a key element of meaningful partnerships in relation to the possible barriers, as well as to the benefits for children. The participants explored effective ways of sharing information about sensitive issues and worked together to reconceptualise the notion of partnership by discussing in small groups, illustrating partnership using markers on a large sheet of paper, and presenting their illustrations and rationales to all groups. Discussions focused on the participants’ perceived elements of effective partnerships, barriers to establishing partnerships and strategies to overcome these. Participants’ ideas were collected during this process, which helped to identify the key characteristics of a partnership model.

Shortly after the partnership sessions took place, all parents and practitioners from the same six settings were asked to complete a post-questionnaire. This helped to identify any differences in the participants’ responses after attending the partnership sessions, as the pre- and post-questionnaires were almost identical, the difference being that the post-questionnaire included an additional question which encouraged participants to provide feedback on the partnership sessions they had attended. The post-questionnaire was not piloted separately, as it was almost identical to the pre-questionnaire, and it was also distributed online (through Survey Monkey). Despite efforts to increase the response rate (e.g., by printing hard copies, delivering, and collecting them in person), only 55 participants (45 parents and 10 practitioners) completed the post-questionnaires, giving a response rate of 20.4%. This was mainly because the post-questionnaire was distributed during the summer, when many participants were away on holiday or parents had moved their children to a different setting.

More than a third (35%) of the participants (28 parents and 6 practitioners) completed both pre- and post-questionnaires, which allowed us to conduct some statistical tests, and 25 (30%) of them also attended both partnership sessions. The rest of the participants completed either the pre or the post questionnaire (but not both), something which was taken into account when conducting any statistical tests during the data analysis.

At the end of the last partnership session, participants were invited to take part in semi-structured interviews. These were conducted with 9 parents, 3 practitioners, and 2 nursery managers, out of the 25 participants that attended the sessions. The interviews were conducted individually (approximately 40 min each) within two months of the final partnership session. The interview questions required the participants to reflect on their experience of attending the two partnership sessions and encouraged them to discuss what they perceived as the key characteristics of an effective partnership model. The interview also encouraged participants to evaluate the impact that participating in the sessions had on their own perceptions of parent-practitioner partnerships and on their practices around this. The interviews provided rich qualitative data and helped to identify individual views in relation to the usefulness and the impact of the partnership sessions.

## Data Analysis

The data analysis occurred in two phases. In the initial phase, the quantitative data obtained from the questionnaires was analysed. The results from the analysis guided the type of data to be collected in the qualitative phase. The aim of the qualitative stage of the study was to further explore and validate the data obtained in the quantitative phase.

For both pre- and post-questionnaires, before any correlation-based analyses were conducted with SPSS, two sets of raw data (parents and practitioners) were extracted from Survey Monkey and analysed in Excel initially to identify any patterns or emerging themes. For the pre-questionnaires, the analysis focused on the existing situation by identifying participants’ perceived benefits and challenges of working in partnership, and the current practices in relation to this. For the post-questionnaires, the focus was on identifying any changes in participants’ perceived benefits and barriers to developing effective partnerships, along with noting any changes in daily practices and attitudes. Meanwhile, it also provided the researchers with further lines of inquiry for one-to-one in-depth interviews.

In relation to the qualitative data analysis, eleven interviews were transcribed using a commercial service. All transcripts were imported into NVivo 11, a qualitative data analysis software package. As Gibbs (2002) argues, the heart of qualitative data analysis is to understand the meaning of the texts. To do that, the interview data was analysed using a typological method suggested by Hatch (2002), which involves nine steps. In the first step, the overall data set is divided into categories, which are derived from the literature review as predetermined typologies. In this case, these were: ‘benefits of partnerships’, ‘barriers of partnerships’ and ‘communication’. After arriving at a set of codes, we searched for the linkages and identified themes and patterns across various data sets. The second step involved reading through the data and findings, classifying and highlighting the evidence in the data relating to the various typologies. The third step entailed developing a summary sheet for each interviewee, which included a brief statement of the evidence identified and categorised by the typology in the second step. The fourth step involved identifying patterns, relationships and themes within the typologies. The fifth step involved identifying data that fit into the theoretical framework developed in the previous step. The sixth step involved identifying excerpts of evidence, which did not fit in the theoretical framework of patterns, relationships, and themes developed in the fourth step. Since this data did not fit into the framework developed, it was not analysed further as part of this study. Finally, although all lived experiences shared by the participants were include in the data analysis, irrespective of the number of occurrences, some key themes were redefined, such as ‘identifying parents’ needs’, ‘funding’, ‘resources’ and ‘training,’ which were derived based on the number of times that were repeated during the interviews. This was decided based on the assumption that the degree of repetition links to degree of importance, and therefore those occurrences that were repeated more than 3 times (from different participants) were considered as key and were grouped together into themes. These findings allowed us to move from breadth to depth and triangulate different views and perceptions from one individual to another and from one method to another.

## Findings and Discussion

The study aimed to identify the key characteristics of an effective partnership model that will promote the development of strong parent-practitioner partnerships in the early years. The partnership sessions provided the space and time for parents and practitioners to come together and identify and explore their perceptions of the key characteristics of an effective partnership model. The pre- and post-session questionnaires, as well as the interviews, provided a substantive amount of quantitative and qualitative data, which helped to understand participants’ lived experiences, comprehend the meaning they made of these experiences, and therefore answer the research questions. The final outcome was the identification of the key features of a partnership model for early years, based on parents’ and practitioners’ perceptions and lived experiences, aiming for a model that can be applied in different contexts. In this part, the focus was on answering the research questions and presenting the key findings, as a means to developing the partnership model.

### Parents and Practitioners’ Perceptions of Working in Partnership and How These Change After Participating at the Partnership Sessions

Findings from the questionnaire suggest that both the parents and the practitioners (80%) are either very interested or extremely interested in practitioner-parent partnerships, with 69% of participants’ interest in practitioner-parent partnerships being higher after the sessions. Furthermore, the post-questionnaire indicated that after the partnership sessions, some settings started recognising the changing demands of family life and trying out new ways of communication, such as social media (Facebook, Twitter), as suggested by Knopf and Swick (2008), while they also looked for additional ways to engage in conversations around the learning taking place in the setting, without necessarily focusing on routines (e.g., eating, sleeping, etc.). The findings suggest that in some settings, managers might feel hesitant to make use of such open access platforms (e.g., social media or other websites), possibly because of previous communication problems experienced in these settings. Nevertheless, the post-questionnaire data analysis reflected that few changes had been noticed by both practitioners and parents within two months of the sessions.

Some interesting and statistically significant differences were identified (with the alpha value set at 0.05) when comparing the responses given by parents to the ones given by practitioners. More specifically, in relation to how practitioners and parents perceived their communication and their own role in developing effective practitioner-parent partnerships, it was interesting to note differences between them in both questionnaires. The pre-questionnaire data analysis suggests that practitioners perceive ‘Sports Day’ (45%) and ‘Fundraising Events’ (65%) as the two most usual ways to involve parents in their children’s learning experiences and to develop the parent-practitioner partnership. Yet, parents did not feel the same, with only 15.73% and 25.84% of them agreeing that ‘Sports Days’ and ‘Fundraising Events’ respectively were the most common ways used to involve parents, demonstrating that perhaps parents and practitioners experience these events differently and may have different values (Hauser-Cram et al., 2003; Vincent, 1996). The same significant gap in the perceptions of ‘Sports Days’ and ‘Fundraising Events’ was also reflected in the post-questionnaire data analysis. This was proven to be a statistically significant difference for both ‘Sports Days’ (Fisher’s Exact Test, p = 0.007) and ‘Fundraising Events’ (Fisher’s Exact Test, p = 0.022).

Both parents (53%) and practitioners (55%) felt that the daily face to face communication or parent meetings were their main communication method. However, 75% of the practitioners considered home visits as a good means of communication, while only 12.4% parents agreed with this, which was a statistically significant difference (Fisher’s Exact Test, p = 0.004). The same pattern of discrepancy was also found in the post-questionnaire (practitioners = 58.33% while parents = 23.08%), which was another statistically significant difference (Fisher’s Exact Test, p = 0.028). Furthermore, the pre-questionnaire data analysis revealed a statistically significant difference between parents and practitioners (Fisher’s Exact test, p < 0.001); while 40% of the practitioners thought the main way of involving parents in the children’s learning experiences was to have parent meetings or face to face chats, only 26% of the parents agreed. In fact, 35% of the parents thought that activities such as ‘Stay and Play’ sessions were more important for them and their children. The same view was shared by 35% of the practitioners for the ‘Stay and Play’ sessions, but at the same time the same percentage of practitioners considered fundraising, fairs or any social events as equally important in involving the parents in children’s learning experiences. These discrepancies between parents and practitioners’ perceptions further highlighted that parents and practitioners have different lived experiences and in some cases expectations (Hauser-Cram et al., 2003; Vincent, 1996). Therefore, communicating, identifying a common starting point, and clarifying these expectations would be beneficial when aiming to develop an effective parent-practitioner partnership.

Looking at ‘Face to Face Meetings’ and the identification of parental needs more closely, it appeared that most parents (55.06%) did not think of this as an effective way of identifying parental needs, even though the vast majority of practitioners (90%) saw this as the main way to identify parents’ needs. Instead, over half of the parents thought that either ‘word of mouth’ or ‘surveys’ were the most common way of identifying parental needs. Besides while many of them (14.61%) felt that there were no specific efforts to identify their needs, the corresponding response from the practitioners was 0%, i.e., all practitioners thought parents’ needs were identified in their practices. This might suggest that when parents and practitioners meet, parents may find it difficult to discuss their specific needs directly with the practitioners, especially if the environment does not allow for privacy (Mapp et al., 2008). Yet, an informal chat or an anonymous/non-direct way of communication, such as surveys, might make it easier for parents to express their thoughts and be more sincere and open about their needs. The post-questionnaire data analysis showed that, while a similar pattern remained, ‘word of mouth’ appeared to have a more important role to play from the perspective of practitioners, the percentage rising from 60% in the pre-questionnaire to 91.67% in the post-questionnaire data analysis, but fewer practitioners selected ‘arranged meeting’, which made the percentage drop from 90 to 66.67% for the post-questionnaire data analysis. One explanation was that casual talks or chats, instead of meetings, were more likely to take place within the time length of the interval between the two questionnaires. This finding highlights the importance of providing a safe and open atmosphere for conversation, with spaces available for parents and practitioners to communicate (Lee, 2006; Mapp et al., 2008).

In addition, practitioners believed that lack of time, both on behalf of parents (40%) and practitioners (35%) was the main barrier to empowering practitioner-parent partnerships. Parents also acknowledged their own lack of time or availability during the settings’ opening hours (66%), rather than the practitioners’ lack of time. Lack of time is a common barrier for parent-practitioner partnerships (Goodall & Montgomery, 2014), however, as some parents and practitioners that participated in the study suggested (10%), this might be because not everybody recognises the positive benefits of such partnerships, which may make them less willing to invest time. This suggests that practitioners may need to be more proactive and to specifically highlight the benefits and importance of partnership, by offering more opportunities for sharing information and encouraging the development of meaningful parent-practitioner partnerships (Wilson, 2015). In the pre-questionnaire, the first choice of main barriers the practitioners chose was "lack of parents' time" which accounted for 90%. Yet, in the post-questionnaire, the first choice changed to "lack of staff time" which accounted for 83.33%. This change might reflect the practitioners' own reflection and a change of attitudes not only towards their own responsibilities but also regarding the way they perceive parents. Interestingly, in the post-questionnaire, parents' first choice had also changed from "parents' lack of availability during setting's working hours" (79.78%) to "lack of staff time" (71.79%) which was also a significant difference (z = 2.12, p = 0.034), possibly indicating a better understanding of the staff’s circumstances (Goodall & Montgomery, 2014; Lee, 2006).

All participants agreed that the partnership sessions provided the space and time for them to discuss and to identify new ways to think about everyday practice and specifically partnerships. Both parents and practitioners appreciated the opportunity to work together and to dedicate time to get to know each other and develop relationships of trust. A parent specifically said, “The biggest thing for me was the chance to engage better with the staff from my own setting. Having the discipline of being outside of the setting and meeting up separately for those the sessions, we’ve never done anything like that before. It’s like meeting these people outside of work and thinking oh, you know, there’s two sides to this”. A practitioner similarly noted that it was beneficial “…being able to talk to other parents that weren’t attending our (setting) but had other experiences from other settings. You realise you’re not alone”.

The findings also indicate that parents and participants share a mutual understanding of the importance that partnerships hold, and both recognised that its effectiveness requires mutual respect and recognition of the contribution each part actively makes towards children’s development (Baum & McMurray-Schwarz, 2004). It is also evident that both sides recognise that effective and meaningful collaboration and communication are essential parts of successful partnerships (Ainscow & Sandill, 2010), and that parent-practitioner partnerships involve having the time and space to reflect, exchange knowledge, and share experiences and ideas and to support the children (Mapp et al. 2008).

### The Key Features of a Parents’ and Practitioners’ Partnership Model

The findings highlighted the importance of effective communication as a two-way process based on dialogue, as well as the importance of trust for both parents and practitioners, something demonstrated through the questionnaire findings as well. During the interviews, one of the parents said that attending the sessions helped her realise that the onus is on parents just as much as on practitioners. As she said: “I used to expect them to come and approach me whereas now I’m much more aware that it’s a two-way thing”, which relates to Goodall and Vorhaus’ (2011, p. 7) comment that “the transfer of knowledge and understanding should be part of a two-way process: not only from school to home but from home to school”. Another parent noticed that their “nursery is emailing more, and they also use other social media” after participating at the partnership sessions. A practitioner also noted that parents started contributing more to their children’s records of learning and share more about the activities they do with their children. These comments suggest a possible positive impact of the partnership sessions to the settings: that when parents and practitioners dedicate time to come together, it is possible to communicate effectively and better recognise each other’s perspectives. In this case, it seems that the practitioners recognised the changing demands on family life and adapted their approaches in terms of communicating with parents, something noted as important by Knopf and Swick (2008). Parents also seemed to have become empowered by realising how important they were in the partnerships’ equation (Rouse, 2012).

Nevertheless, participating in the partnership sessions did not necessarily change participants’ views, but it perhaps helped them think more about their convictions related to partnership. As a mother said, “it was like a Eureka moment, as it was so obvious once it had been said, but until it had been said I hadn’t really thought about it”. A practitioner specifically noted that before attending the sessions, she knew that something was wrong with their approach to working with parents and the kind of feedback given to parents during pick-ups and drop offs but attending the sessions helped her realise that what was lacking was the quality of what was being shared. As she said, “I now need to go back and think, we need to review this… it has actually inspired me to do more at work”, which is in line with Weißenrieder et al. (2015) who note the positive impact of such activities on practitioners’ motivation, confidence and commitment.

This study builds on the findings of previous research that examined the most effective strategies to enrich partnerships, which include adopting a mutual approach by leadership and training (Goodall and Vorhaus 2010), as well as developing mutual trust and respect in relationships, having an open school culture, encouraging strong and valued partnerships and developing useful and easily understood communication (Mutch & Collins, 2012). These factors seem to indicate that long term strategies should recognise the changing demands on family life (Knopf & Swick, 2008) and take a mutual and inclusive approach to the development of values and skills which might be flexible, understanding that communication and dialogue does not work on a ‘one size fits all’ basis (Murray et al., 2015).

Even though, within the existing literature, there is an accepted understanding of the role parents can take, which includes talking, listening, role modelling, managing expectations, and ensuring school attendance (Muschamp et al., 2007), practice often falls short of this ideal (Epstein et al., 2009; Wilson, 2015). This can be the case for a variety of reasons, such as the challenges faced when performance in schools is prioritised over other matters (Rogers, 2007), even when partnerships have a solid theoretical background and are supported both rhetorically and  legislatively. This is often because some educational settings may not promote effective and meaningful partnership opportunities between practitioners and parents (Baum & McMurray-Schwarz, 2004; Phtiaka, 2008). The results of this study support the above and show that barriers to effective partnerships include practitioners’ and parents’ everyday busy lives and routines, indicating the lack of time as well as different interpretations of what an effective partnership is and how it can take place (Pieridou, 2013).

The findings of the study were used to develop a partnership model, with the aim of highlighting the key characteristics of a partnership model. Collaboration and communication were two elements that were discussed in depth during the partnership sessions and were noted as significant by parents and practitioners both in the questionnaires and interviews (Ainscow & Sandill, 2010). Both parents and practitioners agreed that collaboration and communication are key when aiming for a good partnership. In this case, ‘collaboration’ signifies that parents and practitioners work together as equals in an inclusive learning community (Turnbull & Turnbull, 2001). ‘Communication’ represents the importance of the exchanging of information as part of developing a relationship and as part of exploring opportunities to collaborate. Communication can happen at a basic level, but as part of this model it would represent more meaningful discussions including sharing values and beliefs, and developing pedagogy together (Murray et al., 2015).

Another key aspect derived from the findings was the two-way dialogue, something which both parents and practitioners identified as vital in order to keep both sides actively involved in the relationship. As one of the parents said during the interviews, realising the impact of her active engagement and the importance of having a two-way dialogue was revolutionary for her. Being ‘active’ is another relevant characteristic and demonstrates that a partnership is a two-way relationship, involving two-way dialogue, and both parents and practitioners need to be engaged and interested in the partnership for it to work. The findings also highlighted the importance of providing time and space for parents and practitioners to share and develop relationships of trust and respect. A friendly and safe environment in which both groups can actively communicate and collaborate is important, as many stated in both the questionnaires and interviews. Such an environment would enable both sides to develop a relationship and to exchange ideas in a safe and organic way (Murtaza, 2011). Therefore, a ‘friendly’ environment is another characteristic of the partnership model, which emphasises the importance of a non-judgemental, safe environment as well as the importance of confidentiality and anonymity when aiming to develop relationships of trust. Finally, the environment in which this takes place is also important because of the physical and emotional space needed to feel safe and open, with space available to communicate and discuss in confidence (Lee, 2006; Wilson, 2015). Both parents and practitioners highlighted the importance of having the space and time to communicate and develop their partnership.

**Fig. 1:**
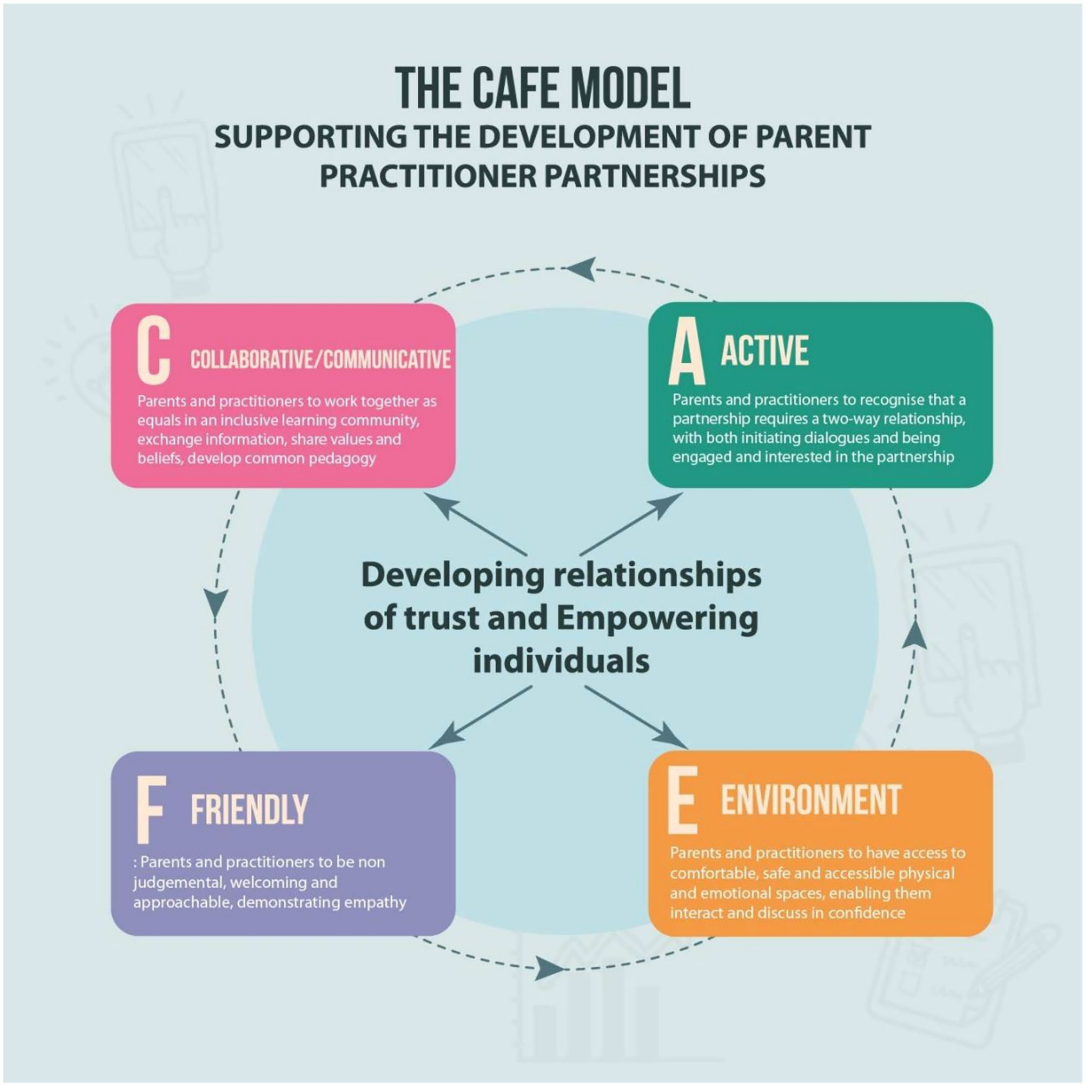
The CAFE model: supporting the development of parent-practitioner partnerships

These characteristics of an effective parent-practitioner partnership model could be summarised with the CAFE acronym which signifies that a Collaborative/Communicative (C), Active (A) and Friendly (F) Environment (E) is essential when aiming to develop effective parent-practitioner partnerships in early years education (see Fig. 1). Such an environment would promote the development of relationships of trust and recognises parents and practitioners as equals, who are working together to ensure best outcomes for the children. This is a key aspect of the CAFE model, especially since previous models, such as the family-centre model (Dunst et al., 1988), saw practitioners as the experts and parents as those in need of support, which increases pressure on practitioners and diminishes the expertise that parents bring to the equation. The CAFE model envisions parents and practitioners as equals and empowers both parties by recognising their strengths and seeing them as equally important in the partnership and in their role relating to the child’s learning and development. With the CAFE model in place, parents and practitioners can be empowered and develop relationships of trust, which would help to sustain and further develop the partnership between them.

These findings illustrate not only the contrasts between the pre-questionnaire responses of parents and practitioners, but also highlight a deeper shared understanding of the value of a two-way relationship. When examining the findings, it becomes clearer that there is a resonance with Hoover-Dempsey and Sandler’s model (2005), as adapted by Hoover-Dempsey et al. (2005), which emphasises the importance of initiating opportunities to become involved in a way that recognises time poverty and strives to find mutually convenient opportunities. Hoover-Dempsey and Sandler’s framework (Hoover-Dempsey et al, 2005) focuses on three motivational elements for affecting parental involvement which include (1) parents’ own beliefs and sense of self-efficacy, (2) parents’ understanding of their importance to the setting and to their children, and (3) their capacity to become involved, in terms of time, skills and knowledge. The invitations that might be extended to parents, by settings, to engagements based on the CAFE model conform to the second, and the findings from this study and further invitations to engage may act as further motivation for the first point, especially when parents’ responses are reflected upon and form the basis of further communications. The CAFE model also builds on the third point, highlighting that this must be a two-way relationship which requires the provision of a safe space, whether that is virtual or face to face.

The CAFE model can also be considered in the context of Epstein’s model of parental involvement, which identifies six types of parental involvement, considered to be influential on children’s outcomes (Epstein et al., 2009), such as parenting, communicating, and learning at home. Whilst the typology of Epstein’s behaviours (Epstein et al., 2009) might be considered limited in terms of understanding the breadth of styles of parental involvement, one aspect shared with the CAFÉ model is the importance of communication. This too is present within the Hoover-Dempsey framework (2005) which heightens the relevance of the research findings and thereby shares a consistent message that effective collaboration and communication can drive strong partnerships.

## Conclusion

The literature review outlines the ongoing and crucial role that parents play in their child’s education, as well as the benefits and the importance of parent-practitioner partnerships on children’s learning and development. This issue has become even more important during the COVID-19 pandemic, a time when a good partnership between parents and practitioners has the potential to play a key role in ensuring that children continue to receive and have access to quality experiences and education (DfE, 2021; Wilson & Waddell, 2020), but which now may have to be mediated through online/remote means that may also be affected by individual’s socioeconomic status (Montacute & Cullinane, 2021; Wilson & Waddell, 2020). The literature also indicates that partnerships are important to counteracting disadvantage, but so far, little is known about how to facilitate partnerships in practice (Khan, 2014; Wilson, 2015). This is especially important given that the early years are varied and transient, thus requiring partnership models to be flexible and localised (Barton et al., 2004; Cottle & Alexander, 2013; Goodall & Montgomery, 2014). It is our view that partnerships in early years need to be examined and re-examined, because circumstances and parental experiences change with time, as do the circumstances and experiences of nurseries and practitioners.

In this study, we worked with parents and practitioners and identified the key characteristics of an effective partnership. The mixed methods approach which, in a unique way, engaged both parents and practitioners as participants, facilitated a better understanding of what both groups perceive as the key characteristics of an effective partnership, based on their own experiences. The partnership sessions provided a platform for thinking together about the importance of partnership and the elements of the partnership CAFE model were subsequently developed. It seems that the experience in the sessions was enriched because parents and practitioners came from a range of settings, which highlighted a more outward facing approach (Goodall & Vorhaus, 2011). By focusing specifically on the partnership, participants were able to value the engagement and commitment demonstrated and develop a more reflective and empathetic perspective to their counterparts.

The findings revealed that both parents and practitioners agree that there are some key ideas that a partnership model should incorporate, which would lead to creating a collaborative, communicative, active, and friendly environment that would recognise them as equals and promote the development of trust. Leading on from this, tasks to build partnerships could be identified as:

1. Reflecting on how well practitioners know and understand the needs of the parents with whom they are working.
2. Identifying suitable methods to improve awareness and communication.
3. Addressing the challenges of time poverty, either by finding more effective ways to enable parents to become involved, or more effectively communicating the importance of parental involvement so that parents feel more motivated to invest their time in this.
4. Reviewing direction of travel of communication: ensuring that practitioners are listening to the diverse needs of parents and valuing their comments, to develop trust.

The above led to the development of the CAFE model, which recognises parents and practitioners as equals that can work together and empower each other in the process, recognising their strengths and weaknesses and supporting each other while supporting the children. The CAFE model is flexible and contemporary and could be used to support the development of effective parent-practitioner partnerships in different contexts, while considering the need for stability and sensitivity of interaction between practitioner and parents. The CAFE model offers a reflective framework whereby some of the main findings of this study can indeed be explored further. This is particularly true of the ‘E’ (environment) element, as this has been altered notably by the influence of COVID-19. It is possible that because of attending the partnership sessions, parents and practitioners then tried to communicate more regularly and more meaningfully, which is a key aspect of effective partnerships that they themselves noted during the sessions. The need for further sessions and other opportunities for the two parties to work together and build partnerships is apparent, and clearly points to the necessity for similar activities to take place in the future. The question of whether this might improve children’s outcomes in any way (See & Gorard, 2013) could be followed up via the application of the CAFE model to investigate parent-practitioner partnerships, for example through a longitudinal analysis into whether the meetings resulted in positive relationships leading to, for example, increased support and signposting to wider services (Khan, 2014).

One of the main drivers for this study was the focus on parental perceptions and employing a methodological approach that brought parents and practitioners from different settings together. This approach highlighted the importance of parents and practitioners working together as equal partners, empowering them as individuals and encouraging the development of strong and valued partnerships (Goodall & Vorhaus, 2011; Mutch & Collins, 2012). For this study, the value was on parents and practitioners working together to identify and develop a partnership model that works for both, the CAFE model. This model helps to address the gap between theory and practice in terms of unpicking the key features of a partnership approach as captured through the lived experience of both parents and practitioners. Future research could focus on evaluating the adoptability of this model and the benefits of employing it as part of a child centred pedagogical approach. It could also focus on developing the model further, in a way that it would be used to evaluate and steer the development of existing partnership approaches (e.g. as part of Ofsted inspections or internal reviews of practice). The model could also be used by nurseries and settings as a guide when developing their ‘working with parents’ ethos as well as their partnership policies.

The key original contribution of this study is the partnership model presented above, which promotes the establishment of strong parent-practitioner partnerships in the early years. Building on Froebelian principles that highlight the importance of family and community (Brehony, 2009), the partnership model explored the importance of collaboration and communication as part of a two-way dialogue. The aim was to identify the key characteristics of a partnership model that would encourage more interest and commitment to partnership. Settings that have done this successfully in the past are those consistently reinforcing the fact that “parents matter”. Such settings develop a two-way partnership with parents based on mutual trust, respect and a commitment to improving learning outcomes (Harris & Goodall, 2007). The CAFE model addresses the gap in the literature in terms of unpicking the key features of a partnership approach as captured through the lived experience of both parents and practitioners.

## Interview Questions:

1. **Background:** Have you attended the Continuous Professional Development programme (I will refer to this from now onwards as CPD) as a parent/carer or as a practitioner? Please explain the reason(s) for your participation.
2. How would you say that a normal day goes on in your life?
  *If teacher:* What ages do you work with? How long have you been working in the early years sector for? Are you enjoying your work?
  *If parent: * How many children do you have? What age is your child that goes to X school? Do you spend some time together discussing school?
3. **Froebel:** As we’ve already stated, this project is funded by Froebel Trust, an organisation in the memory and honour of Frederick Froebel.
  - Have you heard of him before?
    Froebel’s pedagogy puts the child in the centre and recognises the importance of the relationships of every child to family, community and nature, culture and society. The role of play and creativity is also at the centre of Froebel’s pedagogy and these are the integrating elements in development and learning. For Froebel every child is unique and education should aim for the holistic development and overall well-being of every child.
  - What does pedagogy mean to you? Would you say that you agree with Froebel’s view?
4. **Parental Involvement:** What does 'working in partnership with parents/teachers' mean to you? Please answer in your own words.
5. Can you give any examples of when 'partnership with parents' has worked effectively? Can you give any examples of when it has not worked effectively? Why did you think this was?
  What acted as an enabler/ barrier in your collaboration in that instance?
6. What words would you use to describe your experience of partnership with parents/ teachers so far?  Choose as many as you like.
7. **Benefits:** What do you think the benefits of Parental Involvement are?
  Please share your ideas about the benefits of parental involvement in relation to: (a) the setting, (b) the parents, (3) the children, (4) the teacher.
8. **Barriers to Parental Involvement:** Overall, would you say there are some key barriers to parental involvement? What do you think these are? Please feel free to draw on your own experience and provide examples.
  - What would you say are the factors that challenge ‘partnerships with parents’?
9. **Reducing Barriers:** What might reduce these barriers and make it easier for parents to be more involved in their children’s education? What changes do you think would allow teachers to encourage parents to be more involved?
  - Can you make any particular suggestions to improve partnership work?
10. **Maintaining links:** Apart from the teachers’ and parents’ role, how do you think that the setting influences/ maintains partnerships with parents?
  - What do you think makes a setting feel 'parent-friendly'?
  - What support, if any, do you think the setting needs in order need to develop the collaboration with parents?
  - Do you think your setting offers this kind of support currently?
11. **CPD Programme:** Based on your experience, what would you say were the most useful aspects of the CPD programme?
  - What was the impact, if any, of the CPD programme in relation to parental involvement,?
  - How could the CPD programme be improved?
  - Would you recommend this programme to other parents/ teachers/ schools?
12. **Any Other Comments:** Is there anything you would like to add?

## Footnotes

1 When referring to parents we also include carers, and similarly when referring to practitioners we include early years teachers or other professionals working with children in the early years, while by referring to settings we also include schools that work with children in the early years.

## References

[CR1] Ainscow M, Sandill A (2010). Developing inclusive education systems: The role of organisational cultures and leadership. International Journal of Inclusive Education.

[CR2] Australian Children’s Education and Care Quality Authority (ACECQA). (2012). National Quality Framework. Retrieved 29 October 2019, from https://www.acecqa.gov.au/nqf/national-quality-standard

[CR3] Ball, S. J. (1994). *Educational reform: A critical and post-structural approach*. Open University Press.

[CR4] Barnes C (1994). Institutional discrimination, disabled people and interprofessional care. Journal of Interprofessional Care.

[CR5] Barton AC, Drake C, Perez JG, St-Louis K, George M (2004). Ecologies of parental engagement in urban education. Educational Researcher.

[CR6] Barton, L., & Armstrong, F. (2001). Disability, education, and inclusion: Cross-cultural issues and dilemmas. In G. L. Albrecht, K. Seelman, & M. Bury (Eds.), *Handbook of disability studies.* (pp. 693–710). Sage Publications.

[CR7] Baum AC, McMurray-Schwarz P (2004). Preservice teachers' beliefs about family involvement: Implications for teacher education. Early Childhood Education Journal.

[CR8] Boutskou E (2007). Constructing the ‘parents’ in primary schools in Greece: Special education teachers’ whispers. International Journal about Parents in Education.

[CR9] Brehony KJ (2009). Transforming theories of childhood and early childhood education: Child study and the empirical assault on froebelian rationalism. Paedagogica Historica.

[CR11] Brown, C. (1999). Parent voices on advocacy, education, disability and justice. In K. Ballard (Ed.), *Inclusive education: International voices on disability and justice.* (pp. 28–42). Falmer Press.

[CR13] Campbell C, Dalley-Trim L, Cordukes L (2016). ‘You want to get it right’: A regional Queensland school’s experience in strengthening parent–school partnerships. Australasian Journal of Early Childhood.

[CR14] Cohen, L., Manion, L., & Morrison, K. (2011). *Research methods in education*. (7th ed.). Routledge.

[CR15] Cottle M, Alexander E (2013). Parent partnership and 'quality' early years services: Practitioners' perspectives. European Early Childhood Education Research Journal.

[CR16] Daniel G (2015). Patterns of parent involvement: A longitudinal analysis of family–school partnerships in the early years of school in Australia. Australasian Journal of Early Childhood..

[CR17] Davis, H., Day, C., & Bidmead, C. (2002). *Working in partnership with parents: The parent adviser model*. The Psychological Corporation.

[CR18] de Boer A, Pijl SJ, Minnaert A (2010). Attitudes of parents towards inclusive education: A review of the literature. European Journal of Special Needs Education.

[CR19] Department for Education. (2011). *Teachers’ Standards: Guidance for School Leaders, School Staff and Governing Bodies* Department for Education. Retrieved November 3, 2019, from https://assets.publishing.service.gov.uk/government/uploads/system/uploads/attachment_data/file/665520/Teachers__Standards.pdf

[CR20] Department for Education. (2016). *Educational Excellence Everywhere: Presented to Parliament by the Secretary of State for Education by Command of Her Majesty.* Department for Education. Retrieved November 3, 2019, from https://assets.publishing.service.gov.uk/government/uploads/system/uploads/attachment_data/file/508447/Educational_Excellence_Everywhere.pdf

[CR21] Department for Education. (2017). *Statutory Framework for the Early Years Foundation Stage: Setting the Standards for Learning, Development and Care for Children from Birth to five.”* Department for Education. Retrieved November 3, 2019, from https://www.foundationyears.org.uk/files/2017/03/EYFS_STATUTORY_FRAMEWORK_2017.pdf

[CR22] Department for Education. (2021). What parents and carers need to know about early years providers, schools and colleges” Department for Education. Retrieved February 10, 2021, from https://assets.publishing.service.gov.uk/government/uploads/system/uploads/attachment_data/file/958149/What_parents_and_carers_need_to_know_about_early_years_providers__schools_and_colleges_.pdf

[CR23] Department of Education and Skills. (2007). *Every Parent Matters.”* Nottingham: Department for Education and Skills. Retrieved November 4, 2019, from file:///C:/Users/qb901265/Downloads/Every%20Parent%20Matters.pdf

[CR24] Desforges, C., & Abouchaar, A. (2003). The impact of parental involvement, parental support and family education on pupil achievement and adjustment: A literature review*. Vol. 433.* London: Department for Education and Skills. Retrieved November 3, 2019, from http://good-id-in-schools.eu/sites/default/files/sof_migrated_files/sof_files/impactparentalinvolvment.pdf

[CR25] Devine, F. (2004). *Class practices: How parents help their children get good jobs*. Cambridge University Press.

[CR26] Dunst CJ, Dempsey I (2007). Family-professional partnerships and parenting competence, confidence, and enjoyment. International Journal of Disability, Development and Education.

[CR27] Dunst, C. J., Trivette, C. M., & Deal, A. G. (1988). *Enabling and empowering families: Principles and guidelines for practice*. Brookline Books.

[CR29] Epstein, J. L., Sanders, M. G., Sheldon, S. B., Simon, B. S., Salinas, K. C., Jansorn, N. R., Van Voorhis, F. L., Martin, C. S., Thomas, B. G., Greenfeld, M. D., & Hutchins, D. J. (2009). *School, family, and community partnerships: Your handbook for action*. (3rd ed.). Corwin Press.

[CR64] European Commision. (2014). Proposal for key principles of a quality framework for early childhood education and care. European Commission. Report of the Working Group on Early Childhood Education and Care under the Auspices of the European Commission. Retrieved July 11, 2019, from http://ec.europa.eu/assets/eac/education/experts-groups/2011-2013/ecec/ecec-quality-framework_en.pdf

[CR30] Evangelou, M., Sylva, K., Kyriacou, M., Wild, M., & Glenny, G. (2009). Early years learning and development: Literature review. Research Report No. DCSF-RR176. *Department for Children, Schools and Families*. Retrieved October 29, 2019, from https://dera.ioe.ac.uk/11382/2/DCSF-RR176.pdf

[CR31] Foot H, Howe C, Cheyne B, Terras M, Rattray C (2002). parental participation and partnership in pre-school provision. International Journal of Early Years Education.

[CR33] Froebel, F. (1912). *Froebel’s chief writings on education* (S. S. F. Fletcher and James Welton, Trans.). Edward Arnold.

[CR34] Gibbs, G. (2002). *Qualitative data analysis: Explorations with NVivo*. Open University Press.

[CR35] Goodall J, Montgomery C (2014). Parental involvement to parental engagement: A continuum. Educational Review.

[CR36] Goodall, J., & Vorhaus, J. (2011). Review of best practice in parental engagement*.* Research Report DFE-RR 156 Department for Education*.* Retrieved June 21, 2019, from https://dera.ioe.ac.uk/11926/1/DFE-RR156.pdf

[CR37] Harris, A, & Goodall, J. (2007) Engaging parents in raising achievement do parents know they matter? Research Report DCSF-RW004 *Department for Children, Schools and Families*. Retrieved February 21, 2019, from https://dera.ioe.ac.uk/6639/1/DCSF-RW004.pdf

[CR38] Hatch, J. A. (2002). *Doing qualitative research in education settings*. State University of New York Press.

[CR39] Hauser-Cram P, Sirin SR, Stipek D (2003). When teachers' and parents' values differ: Teachers' ratings of academic competence in children from low-income families. Journal of Educational Psychology.

[CR40] Hodge C, Runswich-Cole K (2008). Problematising parent–professional partnerships in education. Disability & Society.

[CR41] Hoover- Dempsey KV, Sandler HM (2005). Why do parents become involved in their children’s education?. Review of Educational Research.

[CR42] Hoover-Dempsey KV, Walker JM, Sandler HM, Whetsel D, Green CL, Wilkins AS, Closson K (2005). Why do parents become involved? Research findings and implications. The Elementary School Journal.

[CR43] Hughes P, MacNaughton G (2002). Preparing early childhood professionals to work with parents: The challenges and diversity and dissensus. Australian Journal of Early Childhood.

[CR44] Khan, L. (2014). *Wanting the best for my children: parents’ voices.* Report for Centre for Mental Health. London: Centre for Mental Health. Retrieved from https://www.centreformentalhealth.org.uk/sites/default/files/2018-09/wantingthebest.pdf

[CR45] Knopf HT, Swick KJ (2008). Using our understanding of families to strengthen family involvement. Early Childhood Education Journal.

[CR46] Lareau A, Adia-Evans S, Yee A (2016). The rules of the game and the uncertain transmission of advantage: Middle-class parents' search for an urban kindergarten. Sociology of Education.

[CR47] Lee JS (2006). Preschool Teachers’ shared beliefs about appropriate pedagogy for 4-year-olds. Early Childhood Education Journal.

[CR48] Mapp KL, Johnson VR, Strickland CS, Meza C (2008). High school family centers: Transformative spaces linking schools and families in support of student learning. Marriage & Family Review.

[CR49] Melhuish EC, Phan MB, Sylva K, Sammons P, Siraj-Blatchford I, Taggart B (2008). Effects of the home learning environment and preschool center experience upon literacy and numeracy development in early primary school. Journal of Social Issues.

[CR50] Ministry of Education. (2017). Te Whariki: Early childhood curriculum. *The Ministry of Education, New Zealand.* Retrieved October 10, 2019, from https://tewhariki.tki.org.nz/assets/Uploads/files/Te-Whariki-Early-Childhood-Curriculum.pdf#page=19

[CR51] Montacute, R. & Cullinane, C. (2021). Learning in lockdown. Retrieved February 10, 2021, from https://www.suttontrust.com/wp-content/uploads/2021/01/Learning-in-Lockdown.pdf

[CR52] Murray E, McFarland-Piazza L, Harrison LJ (2015). Changing patterns of parent-teacher communication and parental involvement from preschool to school. Early Childhood Development and Care.

[CR53] Murray MM, Mereoiu MM (2015). Teacher-parent partnership: An authentic teacher education model to improve student outcomes. Journal of Further and Higher Education.

[CR54] Murtaza KF (2011). Developing child friendly environment in early childhood education classroom in Pakistan. International Journal of Academic Research in Business and Social Sciences.

[CR55] Muschamp, Y., Wikeley, F., Ridge, T., & Balarin, M. (2007). Parenting, caring and educating. In R. Alexander, C. Doddington, J. Gray, L. Hargreaves, & R. Kershner (Eds.), *Cambridge primary review research surveys.* (pp. 103–116). University of Cambridge Faculty of Education.

[CR56] Mutch C, Collins S (2012). Partners in learning: Schools' engagement with parents, families, and communities in New Zealand. School Community Journal.

[CR57] Nachshen JS (2004). Empowerment and families: Building bridges between parents and professionals, theory and research. Journal on Developmental Disabilities.

[CR58] National Council for Teaching and Leadership. (2013). Teachers’ Standards (Early Years) from September 2013. *National College for Teaching and Leadership*. Retrieved May 1, 2018, from https://www.gov.uk/government/uploads/system/uploads/attachment_data/file/211646/Early_Years_Teachers__Standards.pdf

[CR28] Office for Standards in Education, Children's Services and Skills (Ofsted). (2018). Early years inspection handbook. *Office for Standards in Education, Children's Services and Skills (Ofsted)*. Retrieved December 1, 2018, from https://assets.publishing.service.gov.uk/government/uploads/system/uploads/attachment_data/file/760110/EY_inspection_handbook_281118.pdf

[CR59] Organization for Economic Cooperation and Development (OECD). (2017). *PISA 2015 results (Volume III): Students’ Well-being*. OECD Publishing.

[CR60] Phtiaka H (2004). Home school relationships in special and mainstream schools. Educational Review.

[CR61] Phtiaka, H. (2008). The inclusion of children with special needs in mainstream schools through the eyes of the parents: Success, problems and cooperation between family and school, in Phtiaka, H. (Eds.). (2008). *Drop by for a coffee. School-home relationships at the edge of diversity*, pp. 123–168, Athens: Taxideutis [In Greek: Φτιάκα, Ε. 2008. Η ένταξη των παιδιών με ειδικές ανάγκες στο γενικό σχολείο μέσα από τα μάτια των γονιών: Επιτυχίες, προβλήματα και συνεργασία ανάμεσα στην οικογένεια και το σχολείο, στο Φτιάκα, Ε. (επιμ.) 2008. *Περάστε για ένα καφέ*, Αθήνα: Ταξιδευτής.]

[CR62] Pieridou, M. (2013). Special and inclusive education in Cyprus: Case study of a school unit with regards to the implementation of the 113(I)/99 law in educational practice. PhD Thesis, University of Cyprus

[CR63] Pieridou, M, & Phtiaka, H. (2011). *Professionals and parents of children with disabilities in primary schools in Cyprus: Cooperation or conspiracy?* Proceedings of the Hellenic Education Society Conference “Education of children with special needs: A challenge for school and society”, Thessaloniki: University of Macedonia. [In Greek: Πιερίδου, Μ. and Φτιάκα, Ε. (2011), Ειδικοί και γονείς παιδιών με αναπηρία σε γενικά σχολεία της Κύπρου: Συνεργασία ή συνομωσία;, Πρακτικά συνεδρίου της Παιδαγωγικής Εταιρείας Ελλάδος, ‘Εκπαίδευση παιδιών με ειδικές ανάγκες: Μια πρόκληση για το σχολείο και την κοινωνία’, Θεσσαλονίκη: Πανεπιστήμιο Μακεδονίας.]

[CR65] Rogers C (2007). Experiencing an 'inclusive' education: Parents and their children with 'special educational needs'. British Journal of Sociology of Education.

[CR66] Rouse E (2012). Partnerships in early childhood education and care: Empowering parents or empowering practitioners. Global Studies of Childhood.

[CR67] Sammons, P., Toth, K., & Sylva, K., (2015). Background to success: Differences in A-level entries by ethnicity, neighbourhood and gender. *University of Oxford Department of Education*. https://www.suttontrust.com/wp-content/uploads/2015/11/Background-to-Success-Final-1.pdf

[CR68] See, B. H., & Gorard, S. (2013). Do parental involvement interventions increase attainment? A review of the evidence. *Nuffield Foundation.* https://www.nuffieldfoundation.org/sites/default/files/files/Do_parental_involvement_interventions_increase_attainment1.pdf

[CR69] Singh NN (1995). In search of unity: Some thoughts on family–professional relationships in service delivery systems. Journal of Child and Family Studies.

[CR70] Sylva, K., Melhuish, E., Sammons, P., Siraj-Blatchford, I., & Taggart, B. (2004). The effective provision of pre-school education (EPPE) project technical paper 12: The Final Report-Effective Pre-School Education. *The Institute of Education University of London*. Retrieved October 29, 2019, from https://discovery.ucl.ac.uk/id/eprint/10005308/1/EPPE12Sylva2004Effective.pdf

[CR71] Thompson L, Lobb C, Elling R, Herman S, Jurkiewicz T, Hulleza C (1997). Pathways to family empowerment: Effects of family-centered delivery of early intervention services”. Exceptional Children.

[CR72] Turnbull, A., & Turnbull, H. R. (2001). *Families, professionals and exceptionality: Collaborating for empowerment*. (4th ed.). Merrill Prentice Hall.

[CR73] U. S. Department of Health and Human Services Office of Head Start. (2016). *Head Start Program Performance Standards 45 CFR Chapter XIII September 2016.* Washington, DC: Administration for Children and Families. Retrieved January 29, 2018, from https://eclkc.ohs.acf.hhs.gov/sites/default/files/pdf/hspps-appendix.pdf

[CR74] UNESCO. (1994). *The Salamanca statement and framework for action on special needs education*. UNESCO.

[CR75] Vincent, C. (1996). *Parents and teachers, power and participation*. Falmer Press.

[CR76] Vislie L (2003). From integration to inclusion: Focusing global trends and changes in the western European Societies. European Journal of Special Needs Education.

[CR77] Ware LP (1994). Contextual barriers to collaboration. Journal of Educational and Psychological Consultation.

[CR78] Ware, L. P. (1999). My kid and kids kinda like him. In K. Ballard (Ed.), *Inclusive education: International voices on disability and justice.* (pp. 54–77). Falmer Press.

[CR79] Weißenrieder J, Roesken-Winter B, Schueler S, Binner E, Blömeke S (2015). Scaling CPD through professional learning communities: Development of teachers’ self-efficacy in relation to collaboration. ZDM Mathematics Education.

[CR80] Wheeler, H., & Connor, J. (2009). *Parents, early years and learning: Parents as partners in the early years foundation stage: Principles into practice*. National Children’s Bureau.

[CR81] Wilder S (2014). Effects of parental involvement on academic achievement: A meta-synthesis. Educational Review.

[CR82] Wilson, H., & Waddell, S. (2020). COVID-19 and early intervention: Understanding the impact, preparing for recovery. Retrieved February 10, 2021, from https://www.eif.org.uk/files/pdf/covid-19-services-impact-recovery.pdf

[CR83] Wilson, T. (2015). *Working with parents, carers and families in the early years: The essential guide*. Routledge.

[CR84] Zimmerman MA (1995). Psychological empowerment: Issues and illustrations. American Journal of Community Psychology.

[CR85] Zoniou-Sideri, A., & Nteropoulou-Nterou, E. (2008). Cooperation between school and family: Analysis of the experience of disabled children’s parents, in Phtiaka, H. (Eds.). 2008. *Drop by for a coffee. School-home relationships at the edge of diversity*, pp. 69–86, Athens: Taxideutis. [In Greek: Ζώνιου – Σιδέρη, Α. and Ντεροπούλου-Ντέρου, Ε. (2008). Συνεργασία σχολείου και οικογένειας: Ανάλυση λόγου των εμπειριών γονέων παιδιών με αναπηρίες , στο Φτιάκα, Ε. (επιμ.) 2008. *Περάστε για ένα καφέ*, Αθήνα: Ταξιδευτής.]

